# Supplementary material for: Resident memory T cells are a cellular reservoir for HIV in the cervical mucosa
Source: Nat Commun. 2019 Oct 18;10:4739. doi: 10.1038/s41467-019-12732-2 (PMC6802119; doi:10.1038/s41467-019-12732-2)
Supplement: Supplementary file 1 — Supplementary Information [file 41467_2019_12732_MOESM1_ESM.pdf]

Supplementary Information

Resident memory T cells are the major cellular reservoir for HIV in the cervical mucosa

Cantero-Pérez et al.

Supplementary Figure 1

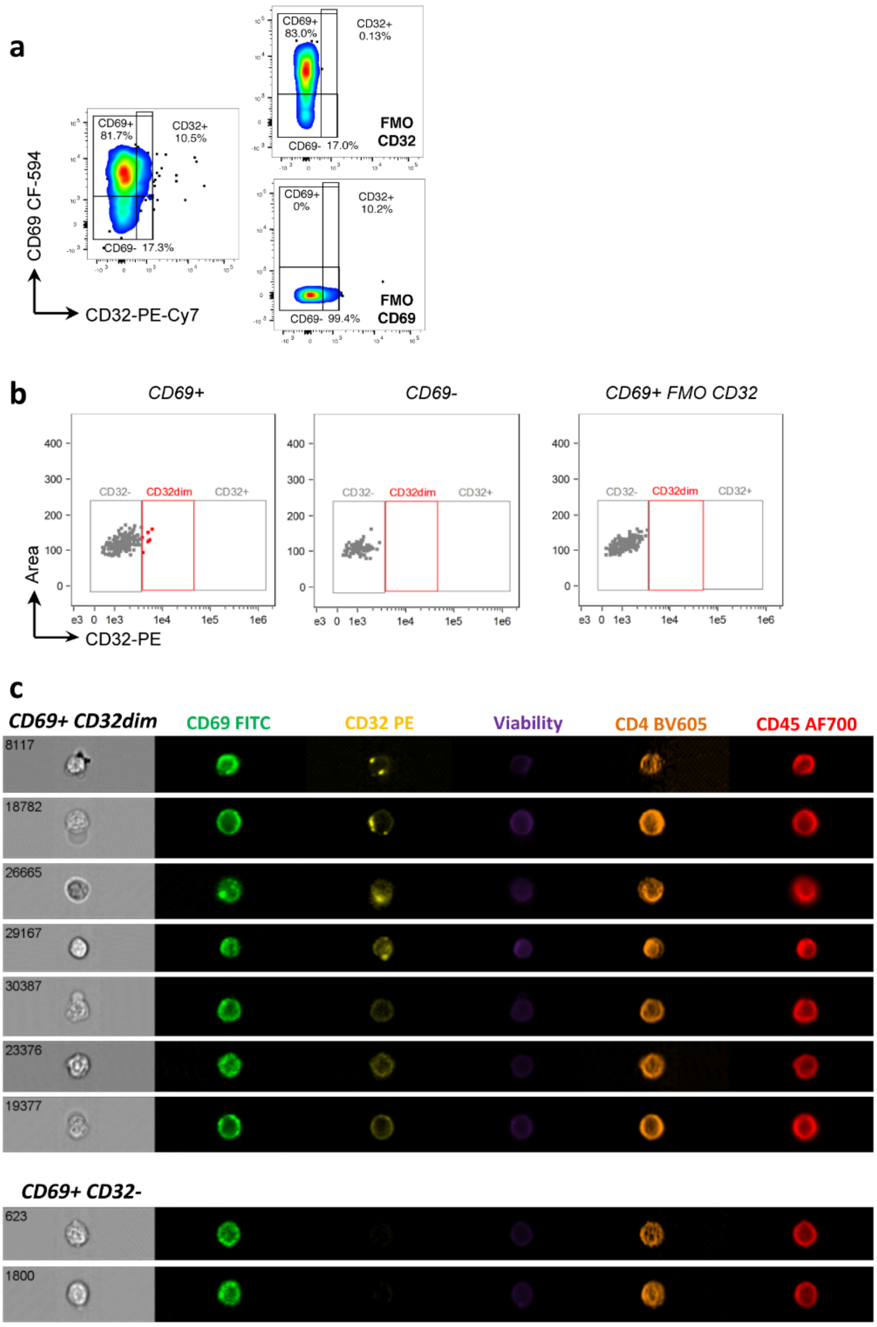

Supplementary Figure 1. CD32-expressing CD4$^+$ T$_{RM}$ controls and flow cytometry imaging. (a) Fluorescence minus one (FMO) controls are shown for CD69 and CD32 markers analyzed by flow cytometry. **(b)** Representative dot plot of the expression of CD32 by CD4$^+$CD69$^{+/-}$ T cells from human cervical tissue of healthy donors analyzed by Amnis® technology. FMO for CD32 expression on CD4$^+$ CD69$^+$ T cells is also shown. **(c)** Representative bright-field and pseudo-colour fluorescence images of CD32^{dim} (up) or CD32$^-$ (down) CD45$^+$CD4$^+$CD69$^+$ T cells imaged using Amnis® technology.

Supplementary Figure 2

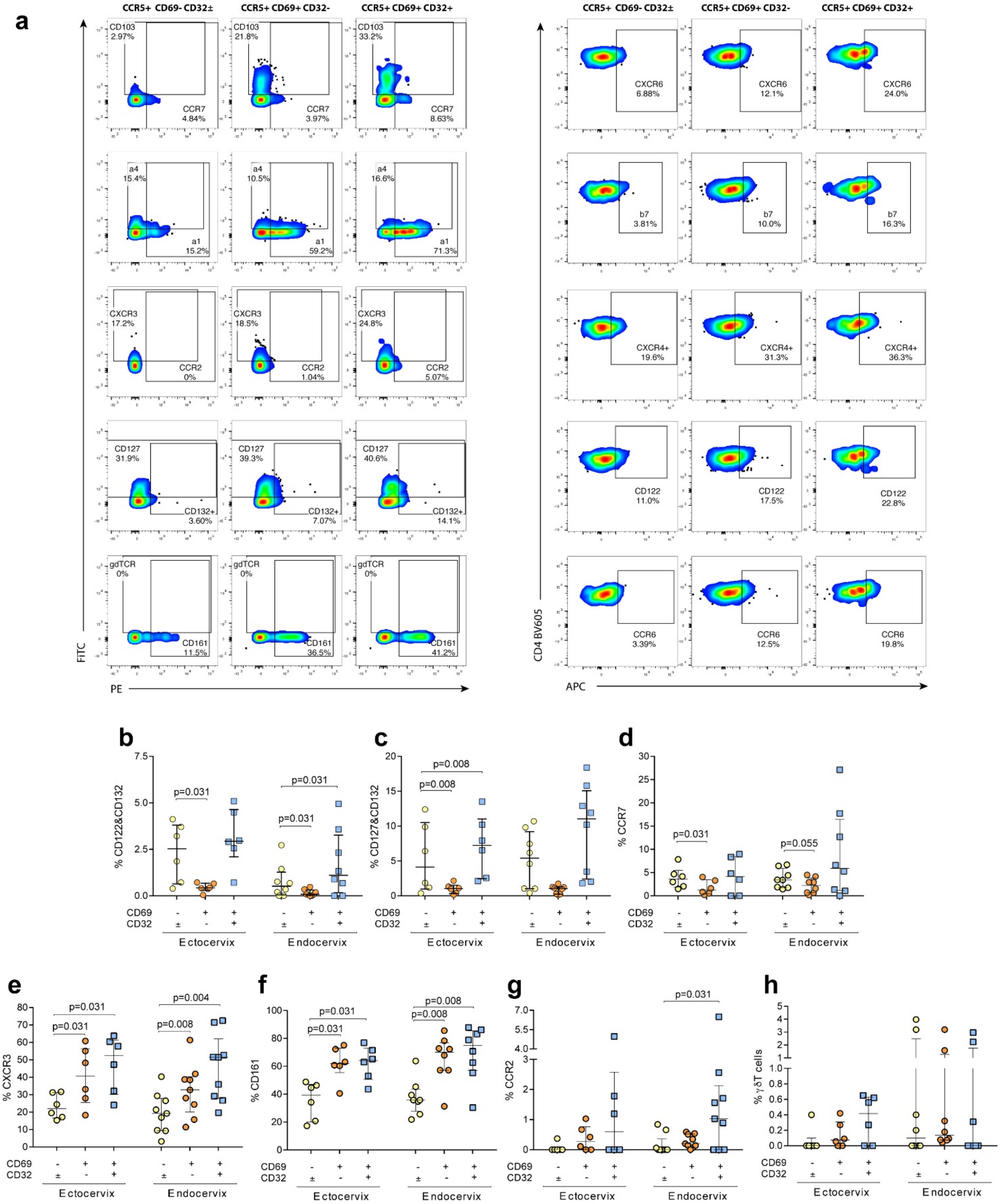

Supplementary Figure 2. CCR5$^+$ CD4$^+$ T$_{RM}$ cell protein expression signature in cervix. (a) Representative flow cytometry plots of the expression of various cell-surface proteins in the three analyzed CD4$^+$CCR5$^+$ T cell subsets from the cervical tissue of healthy donors (from left to right CD69$^-$ non-T$_{RM}$, CD32$^-$ T$_{RM}$ and CD32$^+$ T$_{RM}$). **(b-h)** Frequency of coexpression of interleukins or diverse cell-surface proteins within three different CD4$^+$CCR5$^+$ T cell subsets; namely CD69$^-$ non-T$_{RM}$ (yellow), CD32$^-$ T$_{RM}$ (orange) and CD32$^+$ T$_{RM}$ (blue), in human ectocervix (n=6) and endocervix (n=8 for 2b, 2c, 2d, 2f, 2h; n=9 for 2e, 2g) from healthy donors. Lines and error bars represent median and interquartile ranges. All statistical comparisons were performed using Wilcoxon matched-pairs signed rank test. Source data are provided as a Source Data file.

Supplementary Figure 3

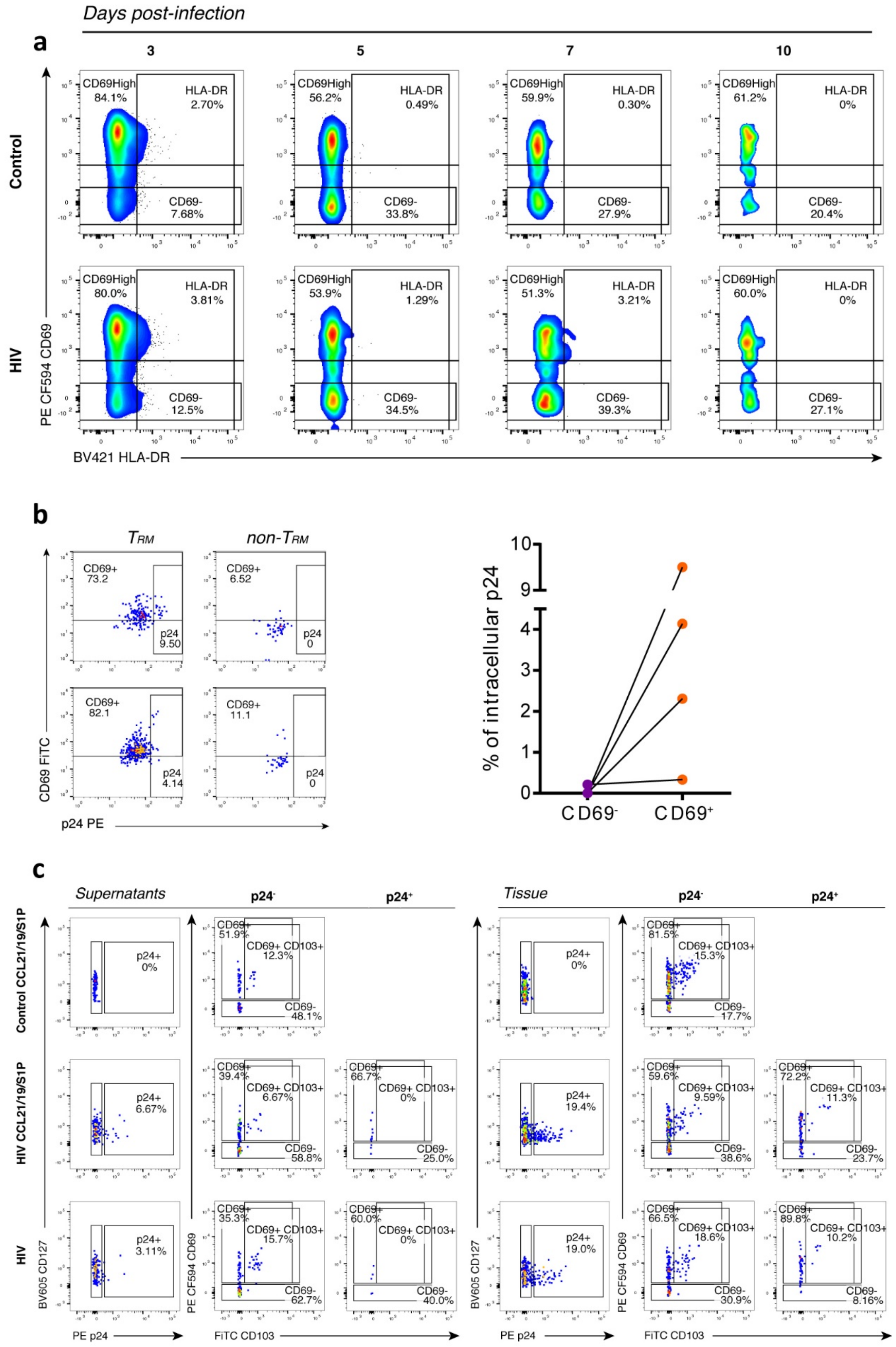

Supplementary Figure 3. CD69 and HLA-DR expression kinetics and tissue retention of HIV infection in the cervical explant model. **(a)** Cervicovaginal tissue blocks were *ex vivo* infected with 7,200 $TCID_{50}$ of an HIV-1_{BAL} and cultured for 3, 5, 7 or 10 days before tissue digestion and flow cytometry analyses. Flow cytometry plots show the frequency of CD69 high, CD69 negative and HLA-DR expression in the non-infected control (up) or HIV-1 *ex vivo* infected (down) $CD4^{+/-}$ T cell gate over time. **(b)** Fresh cervical $CD4^+$ T cells from healthy donors were sorted in $CD69^+$ T_{RM} and $CD69^-$ non-T_{RM} subsets, immediately infected with 7,200 $TCID_{50}$ of an HIV-1_{BAL} and cultured for 3 days before analyzing p24 / CD69 expression as shown in corresponding plots. Data from 2 different donors are shown in the left panel. Adjunct graphic, right panel, shows the frequency of p24 for each of the subsets (n=4). **(c)** Frequency of p24 viral antigen (X axes) *versus* CD127 (Y axes) in total $CD8^-$ T cells from supernatants (left) and corresponding digested tissues (right) treated or not with CCL19, CCL21 and S1P on day 10 of infection. Additional plots show the distribution of the p24 negative or positive cells among $CD69^+$ T_{RM}, $CD69^+$ $CD103^+$ T_{RM} and $CD69^-$ non-T_{RM} phenotypes for each condition. Source data are provided as a Source Data file.

Supplementary Figure 4

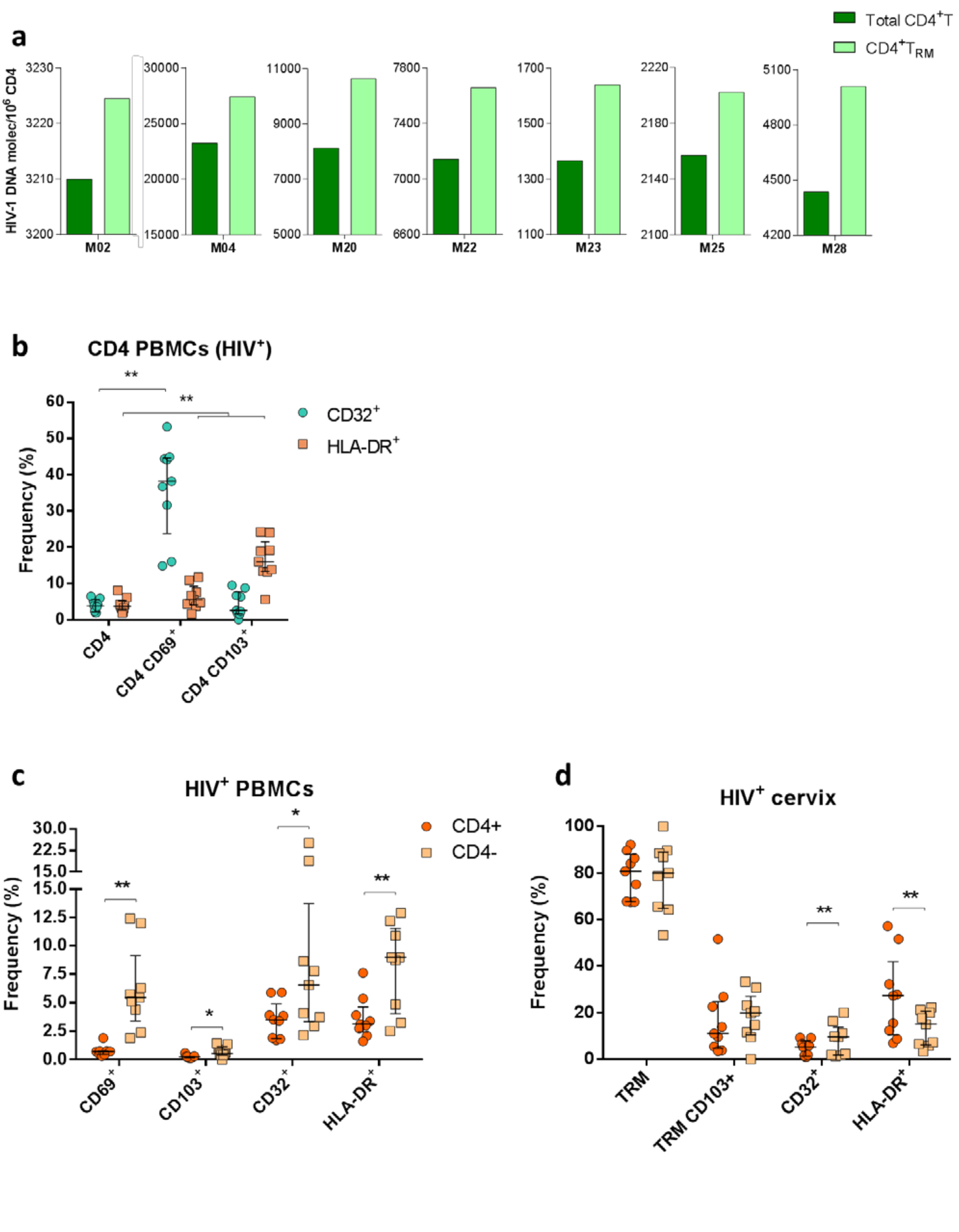

Supplementary Figure 4. vDNA molecules per million of CD4 cells in total CD4$^{+/-}$ vs CD4$^{+/-}$ T$_{RM}$ cells. CD32 and HLA-DR expression in CD4$^{+/-}$ PBMCs from HIV^{+} patients. CD4^{+} vs CD4^{-} T cells phenotype within CD8^{-} T cells from HIV infected patients. (a) Number of vDNA molecules per million of CD4 cells in total CD4$^{+/-}$ vs CD4$^{+/-}$ T$_{RM}$ cells in cervicovaginal tissue from the same 7 HIV^{+} patients showed in *Fig. 5c*. **(b)** Frequency of CD32^{+} and HLA-DR^{+} cells within CD69^{+} or CD103^{+} CD4$^{+/-}$ T cells from blood of HIV^{+} women. Statistical analysis consisted of Wilcoxon matched-pairs signed rank test. **(c)** Frequency of CD69, CD103, CD32 and HLA-DR expression in CD4^{+} T cells and CD4^{-} T cells (not CD8) from blood of ART-treated women (n=9, corresponding to M01-M05, M20, M22-M23 and M25). **(d)** Frequency of CD69^{+} T$_{RM}$, (T$_{RM}$) CD69^{+} CD103^{+} T$_{RM}$, CD32^{+} and HLA-DR^{+} expression in CD4^{+} T cells and CD4^{-} T cells (not CD8) from cervix of the same ART-treated women than in *(c)* (n=9). Lines and error bars display median and interquartile ranges. Statistical analysis consisted of Wilcoxon matched-pairs signed rank test. *$p<0.05$; **$p<0.01$. Source data are provided as a Source Data file.

Supplementary Figure 5

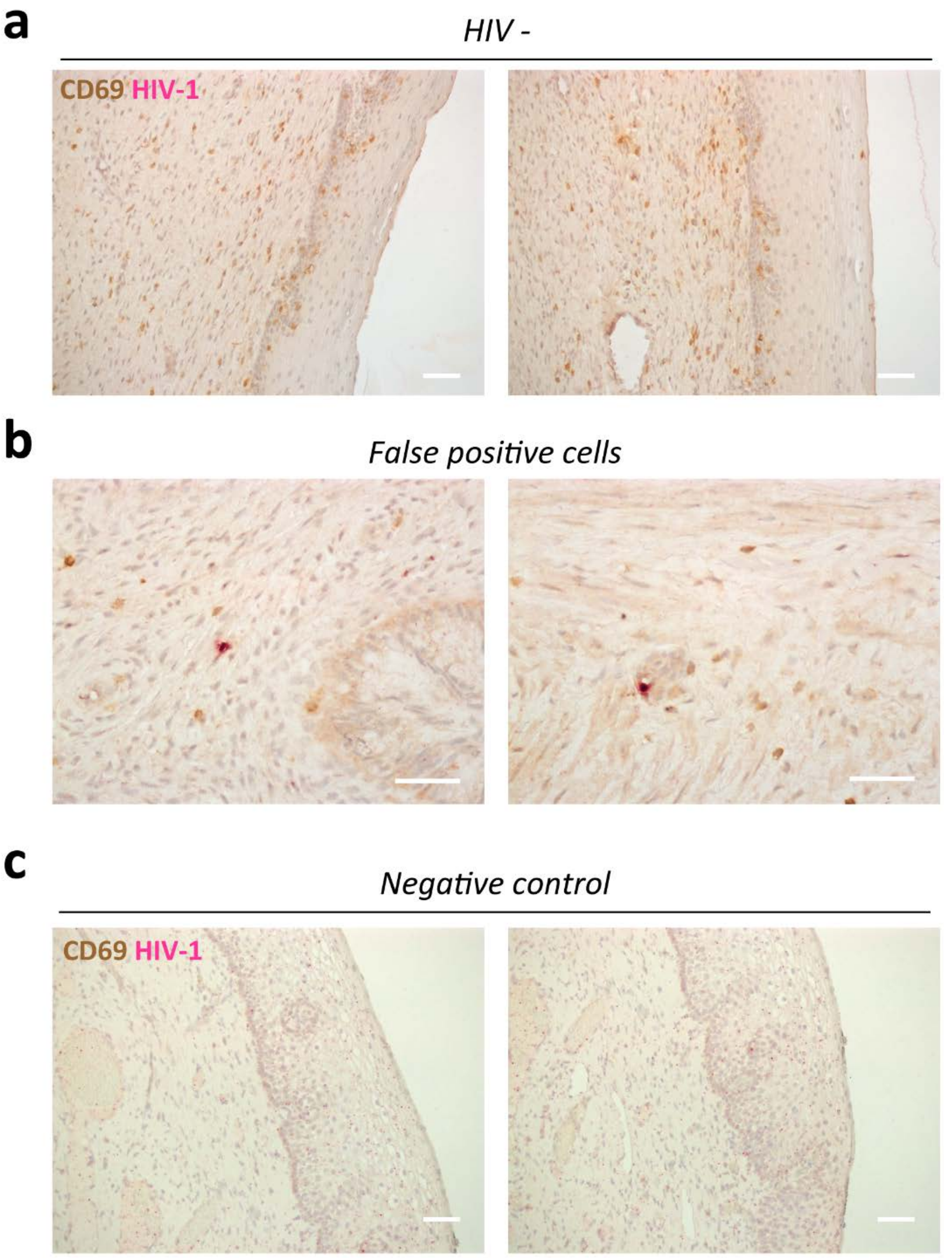

Supplementary Figure 5. RNA *in situ* hybridization and immunohistochemistry controls. Paraffin-embedded cervical tissue from one non-infected and one ART-suppressed HIV-infected women were stained for HIV-1 RNA (pink) using the RNAscope 2.5 HD Duplex Reagent Kit in combination with immunohistochemistry of the CD69 protein (brown). **(a)** Two representative images of an HIV^- donor. Scale bar is 100 µm. **(b).** Two representative images of a cell stained for vRNA in the same HIV^- donor as in (a) (false positive events). Scale bar is 100 µm. **(c)** Negative controls for CD69 staining (no antibody against CD69) and vRNA (no probe against vRNA) in an ART-suppressed HIV-infected patient. Scale bar is 100 µm.

Supplementary Table 1. Number of sorted cells per subset.

Sorting ID	Cervical T_{RM}	Cervical non-T_{RM}	Blood $CD4^+$ T cells $CD69^-$ $CD103^-$	Blood $CD4^+$ $CD69^+$ T cells	Blood $CD4^+$ $CD103^+$ T cells	Blood $CD4^+$ $CD32^+$ T cells
**M00[A]**	NA	NA	NA	NA	NA	NA
**M02**	1,816	218	508,573	8,731	2,588	8,644
**M03**	532	85	500,000	6,894	3,700	7,055
**M04**	556	618	500,000	75,102	25,736	81,149
**M05**	2,110	777	500,000	75,514	25,537	171,805
**M20**	7,928	4,243	500,000	57,893	7,289	2,250
**M22**	4,867	804	500,000	62,340	6,873	8,184
**M23**	3,362	1,605	500,000	51,051	19,058	10,513
**M25**	3,664	492	500,000	170,765	22,306	23,033
**M28**	21,917	5,244	500,000	26,271	7,828	18,883

[A]In this sample only total $CD4^{+/-}$ T cells were sorted obtaining: 36,130 cell from cervix and 94,532 cells from blood.
