# Supplementary material for: Resident memory T cells are a cellular reservoir for HIV in the cervical mucosa
Source: Nat Commun. 2019 Oct 18;10:4739. doi: 10.1038/s41467-019-12732-2 (PMC6802119; doi:10.1038/s41467-019-12732-2)
Supplement: Supplementary file 3 — Reporting Summary [file 41467_2019_12732_MOESM3_ESM.pdf]

nature research

Corresponding author(s): Meritxell Genescà NCOMMS-19-03831

Last updated by author(s): Sep 20, 2019

Reporting Summary

Nature Research wishes to improve the reproducibility of the work that we publish. This form provides structure for consistency and transparency in reporting. For further information on Nature Research policies, see Authors & Referees and the Editorial Policy Checklist.

Statistics

For all statistical analyses, confirm that the following items are present in the figure legend, table legend, main text, or Methods section.

n/a	Confirmed	
☐	☒	The exact sample size (*n*) for each experimental group/condition, given as a discrete number and unit of measurement
☐	☒	A statement on whether measurements were taken from distinct samples or whether the same sample was measured repeatedly
☐	☒	The statistical test(s) used AND whether they are one- or two-sided<br>*Only common tests should be described solely by name; describe more complex techniques in the Methods section.*
☐	☒	A description of all covariates tested
☐	☒	A description of any assumptions or corrections, such as tests of normality and adjustment for multiple comparisons
☐	☒	A full description of the statistical parameters including central tendency (e.g. means) or other basic estimates (e.g. regression coefficient) AND variation (e.g. standard deviation) or associated estimates of uncertainty (e.g. confidence intervals)
☐	☒	For null hypothesis testing, the test statistic (e.g. *F*, *t*, *r*) with confidence intervals, effect sizes, degrees of freedom and *P* value noted<br>*Give P values as exact values whenever suitable.*
☒	☐	For Bayesian analysis, information on the choice of priors and Markov chain Monte Carlo settings
☒	☐	For hierarchical and complex designs, identification of the appropriate level for tests and full reporting of outcomes
☒	☐	Estimates of effect sizes (e.g. Cohen's *d*, Pearson's *r*), indicating how they were calculated

Our web collection on statistics for biologists contains articles on many of the points above.

Software and code

Policy information about availability of computer code

Data collection	No software used.
Data analysis	We used GraphPad Prism (version 7.0) for statistical analysis. For flow cytometry analysis we used FlowJo vX.0.7 (TreeStar), for flow imaging analysis IDEAS v6.1 and for microscopy images analysis ImageJ 1.50i.

For manuscripts utilizing custom algorithms or software that are central to the research but not yet described in published literature, software must be made available to editors/reviewers. We strongly encourage code deposition in a community repository (e.g. GitHub). See the Nature Research guidelines for submitting code & software for further information.

Data

Policy information about availability of data

All manuscripts must include a data availability statement. This statement should provide the following information, where applicable:

- Accession codes, unique identifiers, or web links for publicly available datasets
- A list of figures that have associated raw data
- A description of any restrictions on data availability

There are no restriction for any materials used in this study.

Field-specific reporting

Please select the one below that is the best fit for your research. If you are not sure, read the appropriate sections before making your selection.

☒ Life sciences ☐ Behavioural & social sciences ☐ Ecological, evolutionary & environmental sciences

For a reference copy of the document with all sections, see nature.com/documents/nr-reporting-summary-flat.pdf

Life sciences study design

All studies must disclose on these points even when the disclosure is negative.

Sample size	No sample-size calculations were performed. Based on tissue availability, sample size was determined to be adequate based on the magnitude and consistency of measurable differences between groups.
Data exclusions	Two samples lacking epithelial tissue in the cervical slide were excluded for the ISH/IHC analysis.
Replication	Replicate experiments were successful.
Randomization	Randomization was not relevant to this study.
Blinding	Analyses were unblinded

Reporting for specific materials, systems and methods

We require information from authors about some types of materials, experimental systems and methods used in many studies. Here, indicate whether each material, system or method listed is relevant to your study. If you are not sure if a list item applies to your research, read the appropriate section before selecting a response.

Materials & experimental systems

n/a	Involved in the study	
☐	☒	Antibodies
☐	☒	Eukaryotic cell lines
☒	☐	Palaeontology
☒	☐	Animals and other organisms
☐	☒	Human research participants
☒	☐	Clinical data

Methods

n/a	Involved in the study	
☒	☐	ChIP-seq
☐	☒	Flow cytometry
☒	☐	MRI-based neuroimaging

Antibodies

Antibodies used	All antibodies used in this study are detailed in material and methods section.
Validation	All antibodies are commercially available and were commercially validated.

Eukaryotic cell lines

Policy information about cell lines

Cell line source(s)	J-Lat9.2 cells were obtained from the AIDS Reagent Bank.
Authentication	No authentication was performed.
Mycoplasma contamination	J-Lat9.2 cells were negative for mycoplasma contamination.
Commonly misidentified lines (See ICLAC register)	No commonly misidentified cell lines were used.

Human research participants

Policy information about studies involving human research participants

Population characteristics	HIV- women undergoing non-neoplastic hysterectomies provided cervical tissue for this study (range 26-74 years old). Information about the characteristics of the HIV+ women included in this study is detailed in Table 1. Basically all HIV+ patients were ART-treated with undetectable viral load (< 50copies HIV-RNA/ml plasma), except for one patient with detectable viral load (> 50 copies HIV-RNA/ml plasma).
Recruitment	HIV+ and HIV- donors were recruited based on surgical planification. Women undergoing non-neoplastic hysterectomies were recruited by collaborating gynecologists at Hospital Universitari Vall d'Hebron (HUVH) in Barcelona, Spain. HIV+ women undergoing a hysterectomy or a cone biopsy at the HUVH, at the Germans Trias i Pujol University Hospital (HUGTP) or at the Parc de Salut Mar (Barcelona, Spain) were also recruited by collaborating gynecologists, in which a coded blood sample was also obtained for this study.

Ethics oversight	Study protocols were approved by the corresponding Institutional Review Board (numbers PR (IR)294/2017 for the HUVH, PI-17-159 for the HUGTP and 2018/8017/I for the Parc de Salut Mar) and written informed consent was provided by all patients recruited to this study.

Note that full information on the approval of the study protocol must also be provided in the manuscript.

Flow Cytometry

Plots

Confirm that:

☒ The axis labels state the marker and fluorochrome used (e.g. CD4-FITC).

☒ The axis scales are clearly visible. Include numbers along axes only for bottom left plot of group (a 'group' is an analysis of identical markers).

☒ All plots are contour plots with outliers or pseudocolor plots.

☒ A numerical value for number of cells or percentage (with statistics) is provided.

Methodology

Sample preparation	PBMC were isolated by Ficoll density gradient centrifugation and stained as indicated in materials and methods section. Cervical tissue was digested with an appropiated protocol (indicated in materials and methods section), stained with viability dye, then stained with indicated antibodies and resuspended either in 1X PBS-2%FBS-1mM EDTA for sorting or in 1% paraformaldehyde for fix it before acquisition on an analyzer.
Instrument	BD FACS Fortessa analyzer, BD FACS Aria sorter, BD FACS Calibur analyzer and an Amnis® ImageStreamx.
Software	FACS Diva and CellQuest for data collection. FlowJo and IDEAS software for data analysis.
Cell population abundance	No tests were performed to asses the purity of each population after sorting.
Gating strategy	All samples were initially gated using forward scatter and side scatter to identify events corresponding to cells or, alternatively by CD45 and side scatter to identify events corresponding to hemapoietic cells, and then using forward scatter height vs. area and side scatter height vs. area to enrich for single cells, next alive cells were selected by negativity for viability dye. The follow gating steps are presented in principal and supplementary figures.

☒ Tick this box to confirm that a figure exemplifying the gating strategy is provided in the Supplementary Information.
